# Semi-Supervised Learning in Medical Images Through Graph-Embedded Random Forest

**DOI:** 10.3389/fninf.2020.601829

**Published:** 2020-11-10

**Authors:** Lin Gu, Xiaowei Zhang, Shaodi You, Shen Zhao, Zhenzhong Liu, Tatsuya Harada

**Affiliations:** ^1^RIKEN AIP, Tokyo, Japan; ^2^Research Center for Advanced Science and Technology (RCAST), The University of Tokyo, Tokyo, Japan; ^3^Bioinformatics Institute (BII), A^*^STAR, Singapore, Singapore; ^4^Faculty of Science, Institute of Informatics, University of Amsterdam, Amsterdam, Netherlands; ^5^Department of Medical Physics, Western University, London, ON, Canada; ^6^Tianjin Key Laboratory for Advanced Mechatronic System Design and Intelligent Control, School of Mechanical Engineering, Tianjin University of Technology, Tianjin, China; ^7^National Demonstration Center for Experimental Mechanical and Electrical Engineering Education, Tianjin University of Technology, Tianjin, China

**Keywords:** vessel segmentation, semi-supervised learning, manifold learning, central nervous system (CNS), retinal image

## Abstract

One major challenge in medical imaging analysis is the lack of label and annotation which usually requires medical knowledge and training. This issue is particularly serious in the brain image analysis such as the analysis of retinal vasculature, which directly reflects the vascular condition of Central Nervous System (CNS). In this paper, we present a novel semi-supervised learning algorithm to boost the performance of random forest under limited labeled data by exploiting the local structure of unlabeled data. We identify the key bottleneck of random forest to be the information gain calculation and replace it with a graph-embedded entropy which is more reliable for insufficient labeled data scenario. By properly modifying the training process of standard random forest, our algorithm significantly improves the performance while preserving the virtue of random forest such as low computational burden and robustness over over-fitting. Our method has shown a superior performance on both medical imaging analysis and machine learning benchmarks.

## 1. Introduction

Machine learning has been widely applied to analyze medical images such as an image of the brain. For example, the automatic segmentation of brain tumor (Soltaninejad et al., 2018) could help predict Patient Survival from MRI data. However, traditional methods usually require a large number of diagnosed examples. Collecting raw data during routine screening is possible but making annotations and diagnoses for them is costly and time-consuming for medical experts. To deal with this challenge, we propose a novel graph-embedded semi-supervised algorithm that makes use of the unlabeled data to boost the performance of the random forest. We specifically evaluate the proposed method on both a neuronal image and the retinal image analysis that is highly related to diabetic retinopathy (DR) (Niu et al., 2019) and Alzheimer's Disease (AD) (Liao et al., 2018), and make the following specific contributions:

1. We empirically validate that the performance bottleneck of random forest under limited training samples is the biased information gain calculation.
2. We propose a new semi-supervised entropy calculation by incorporating local structure of unlabeled data.
3. We propose a novel semi-supervised random forest which shows advantage performance of the state-of-the-art in both medical imaging analysis and machine learning benchmarks.

Among various supervised algorithms, random forest or random decision trees (Breiman et al., 1984; Criminisi et al., 2012) are one of the state-of-the-art machine learning algorithms for medical imaging applications. Despite its robustness and efficiency, its performance relies heavily on sufficiently labeled training data. However, annotating a large amount of medical data is time-consuming and requires domain knowledge. To alleviate the challenge of having enough labeled data, a class of learning methods named semi-supervised learning (SSL) (Joachims, 1999; Zhu et al., 2003; Belkin and Niyogi, 2004; Zhou et al., 2004; Chapelle et al., 2006; Zhu, 2006) were proposed to leverage unlabeled data to improve the performance. Leistner et al. (2009) proposed a semi-supervised random forest which maximizes the data margin via deterministic annealing (DA). Liu et al. (2015) showed that the splitting strategy appears to be the bottleneck of performance in a random forest. The authors estimate the unlabeled data through kernel density estimation (KDE) on the projected subspace, and when constructing the internal node, they progressively refine the splitting function with the acquired labels through KDE until it converges. Without explicit affinity relation, CoForest (Li and Zhou, 2007) iteratively guesses the unlabeled data with the rest of the trees in the forest and then uses the new labeled data to refine the tree. Semi-supervised based super-pixel (Gu et al., 2017) has proved to be effective in the segmentation of both a retinal image and a neuronal image.

**Figure 1:**
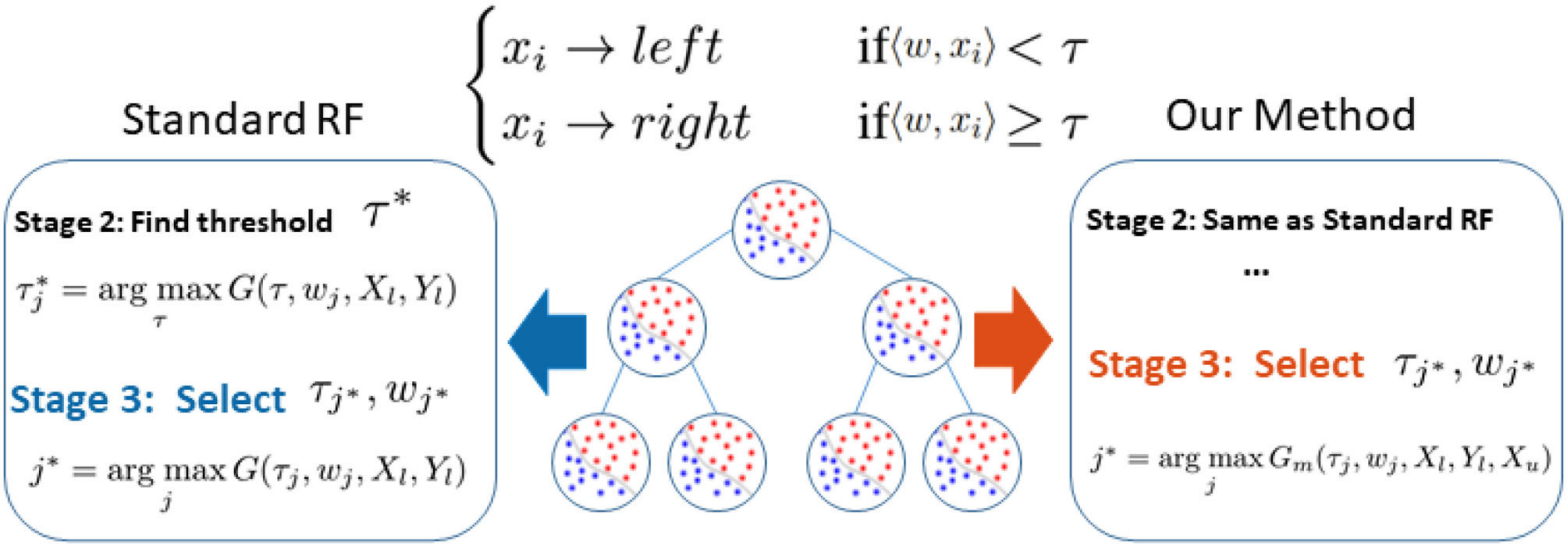
Difference between our method and standard random forest. Noting that the performance bottleneck (shown in blue) is the biased information gain *G*(τ_*j*_, *w*_*j*_, *X*_*l*_, *Y*_*l*_) calculation based on limited labeled data *X*_*l*_, *Y*_*l*_ in Stage 3, we replace *G*(.) with our novel graph-embedded *G*_*m*_(., *X*_*u*_) which considers unlabeled data *X*_*u*_ (shown in red).

Following the research line of a previous semi-supervised random forest (RF), we identify that RF's performance bottleneck, under insufficient data, is the biased information gain calculation when selecting an optimal splitting parameter (shown as blue in Figure 1). Therefore, as illustrated in red in Figure 1, we slightly modified the training procedure of RF to relieve this bias. We replace the original information gain with our novel graph-embedded entropy which exploits the data structure of unlabeled data. Specifically, we first use both labeled and unlabeled data to construct a graph whose weights measure local similarity among data and then minimize a loss function that sums the supervised loss over labeled data and a graph Laplacian regularization term. From the optimal solution, we can get label information of unlabeled data which is utilized to estimate a more accurate information gain for node splitting. Since a major part of training and the whole testing remains unchanged, our graph-embedded random forest could significantly improve the performance without losing the virtue of a standard random forest such as low computational burden and robustness over over-fitting.

## 2. Analysis of Performance Bottleneck

Let us first review the construction of the random forest (Breiman et al., 1984) to figure out why random forest fails under limited training data. A random forest is an ensemble of decision trees: {*t*_1_, *t*_2_, ..., *t*_*T*_}, of which an individual tree is independently trained and tested.

**Training Procedure:** Each decision tree *t*, as illustrated in Figure 1, learns to classify a training sample *x* ∈ X to the corresponding label *y* by recursively branching it to the left or right child until reaching a leaf node. In particular, each node is associated with a binary split function *h*(*x*_*i*_, *w*, τ), e.g., oblique linear split function

(1) $$\begin{array}{r} h\left(x_{i},w,\tau \right)=\left[\langle w,x_{i}\rangle <\tau \right], \end{array}$$

where [.] is an indicative function and τ is a scaler threshold. *w* ∈ *R*^*d*^ serves as a feature weight parameter that projects the high dimension data *x* ∈ *R*^*d*^ to a one dimensional subspace.

Given a candidate splitting function *h*(*x, w*_*j*_, τ_*j*_), its splitting quality is measured by information gain *G*(*w*_*j*_, τ_*j*_). In practice, given the training data *X* and their labels *Y*, the construction of the splitting node, as illustrated in the left side of Figure 1, comprises the following three stages:

**Algorithm 1:** Training of node splitting.

1: Randomly generates a set of feature subspace candidates {*w*_*j*_}
2: For each *w*_*j*_, find the optimal $\tau_{j}^{*}=\underset{\tau }{\text{argmax}}G\left(w_{j},\tau ,X,Y\right)$ that best splits the data.
3: Among all {*w*_*j*_, $\tau_{j}^{*}$}, pick the one with largest information gain: $j^{*}=\underset{j}{\text{argmax}}G\left(w_{j},\tau_{j}^{*},X,Y\right)$

Through the above stages, each split node is associated with a splitting function *h*(*x, w*, τ) that best splits the training data.

**Testing Procedure:** When testing data *x*, the trained random forest predicts the probability of its label by averaging the ensemble prediction as $\hat{p}\left(y|x\right)=\underset{t}{\sum }p_{t}\left(y|x\right)$, where *p*_*t*_(*y*|*x*) denotes the empirical label distribution of the training samples that reach leaf note of tree *t*.

### 2.1. Performance Bottleneck Under Insufficient Data

According to the study of Liu et al. (2015), insufficient training data would impact the performance of RF in three ways (Liu et al., 2015): (1) limited forest depth; (2) inaccurate prediction model of leaf nodes; (3) sub-optimal splitting strategy. Among them, Liu et al. (2015) identified that (1) is inevitable, and (2) is solvable with their proposed strategy. In this paper, we further improve the method by tackling (3).

We claim that the performance bottleneck of random forest is its sub-optimal splitting strategy in Algorithm 1. To empirically support this claim, we build three random forests, similar to Liu et al. (2015), for comparison: the first one, the **Control** is trained with a small size of a training set *S*1 as control; the second one, the **Perfect Stage 3** is constructed with the same training set *S*1 but its node splitting uses a large training set *S*2 to select the optimal parameter in stage 3 of Algorithm 1, to simulate the case that random forest selects the optimal parameter of stage 3 with full information; the third one, the **Perfect Splitting** is constructed with *S*1 while *S*2 was used for both Stage 2 and 3 of Algorithm 1.

**Figure 2:**
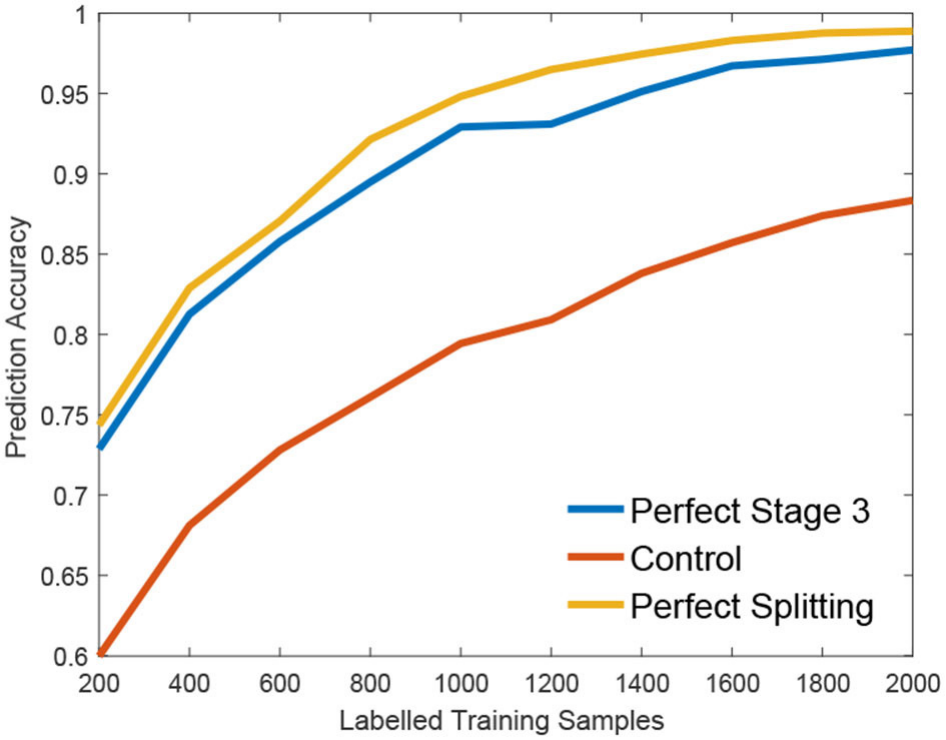
Empirical validation of performance Bottleneck.

Following the protocol of Liu et al. (2015), each random forest comprises 100 trees and the same entropy gain is adopted as the splitting criterion. We evaluate three random forests on Madelon (Guyon et al., 2004), a widely used machine learning benchmark. As shown in Figure 2, Perfect Stage 3, which only uses the full information to select the best parameter set, significantly improves the performance compared to the control group. Interestingly, the Perfect Splitting one, which utilizes the full information for both optimal parameter proposing (Stage 2) and optimal parameter decision (Stage 3), only makes a subtle improvement compared to Perfect Stage 3.

From Figure 2, we found that Stage 3, optimal parameter selection, is the performance bottleneck of the splitting node construction, which is also the keystone of random forest construction (Liu et al., 2015). When deciding the optimal parameter, random forest often fails to find the best one as its information gain calculation *g*(*w*, τ) is biased under insufficient training data. Interestingly, insufficient data has a smaller effect on the Stage 2, parameter proposal. Motivated by this observation, we propose a new information calculation which exploits unlabeled data to make a better parameter selection in Stage 3 of Algorithm 1.

## 3. Graph-Embedded Representation of Information Gain

In the previous section, we show that gain estimation appears to be the performance bottleneck of random forests. Empirically, we show that more label information helps to obtain more accurate gain estimation. This encourages us to consider the possibility of mining label information from unlabeled data through structural connections between labeled and unlabeled data. In particular, we perform a graph-based semi-supervised learning to get label information of unlabeled data, and compute information gain from both labeled and unlabeled data. To achieve a better gain estimation, we embed all data into a graph. Moreover, we assume the underlying structure of all data form a manifold, and compute data similarity based on the assumption.

Let *l* and *u* be the number of labeled and unlabeled instances, respectively. Let $X_{l}={\left[x_{1},\cdots \;,x_{l}\right]}^{⊤}\in {\mathbb{R}}^{d\times l}$ be the matrix of feature vectors of labeled instances, and $X_{u}={\left[x_{l+1},\cdots \;,x_{l+u}\right]}^{⊤}\in {\mathbb{R}}^{d\times u}$ be the matrix of unlabeled instances. To accommodate label information, we define a label matrix *Y* ∈ ℝ^(*l*+*u*) × *K*^ (assuming there are *K* class labels available), with each entry *Y*_*ik*_ containing 1 provided the *i*-th data belongs to *X*_*l*_ and is labeled with class *k*, and 0 otherwise. Besides, we define *Y*_*l*_ as a submatrix of *Y* corresponding to the labeled data, $y_{i}\in {\mathbb{R}}^{K}$ as the *i*-th row of *Y* corresponding to *x*_*l*_, and $y_{l}\in {\mathbb{R}}^{l}$ as the vector of class labels for *X*_*l*_.

Based on both labeled and unlabeled instances, our purpose is to learn a mapping *f* : ℝ^*d*^ → ℝ^*K*^ and predict the label of instance *x* as $k^{*}:=\text{arg}\underset{k}{\max}f_{k}\left(x\right)$. Many semi-supervised learning algorithms use the following regularized framework

$$\overset{l}{\underset{i=1}{\sum }}\text{loss}\left(y_{i},f\left(x_{i}\right)\right)+\lambda \overset{l+u}{\underset{i,j=l+1,i\neq j}{\sum }}s\left(x_{i},x_{j}\right){\left\|f\left(x_{i}\right)-f\left(x_{j}\right)\right\|}_{2}^{2},$$

where *loss*() is a loss function and *s*(*x*_*i*_, *x*_*j*_) is a similarity function. In this paper, we apply the idea of graph embedding to learn *f*. We construct a graph G=(V,E,W), where each node in *V* denotes a training instance and *W* ∈ ℝ^(*l*+*u*) × (*l*+*u*)^ denotes a symmetric weight matrix. *W* is computed as follows: for each point find *t* nearest neighbors, and $W_{ij}=exp\left(-{\left\|x_{i}-x_{j}\right\|}_{2}^{2}/\sigma^{2}\right)$ if (*x*_*i*_, *x*_*j*_) are neighbors, 0 otherwise. Such construction of graph implicitly assumes that all data resides on some manifold and exploits local structure. Based on the graph embedding, we propose to minimize

(2) $$\begin{array}{r} L\left(\left\{f_{i}\right\}\right)=\frac{1}{2}\left(\overset{l+u}{\underset{i=1}{\sum }}\|f_{i}-y_{i}{\|}_{2}^{2}+\lambda \overset{l+u}{\underset{i,j=1}{\sum }}W_{ij}||\frac{f_{i}}{\sqrt{D_{ii}}}-\frac{f_{j}}{\sqrt{D_{jj}}}|{|}_{2}^{2}\right), \end{array}$$

where *D* is a diagonal matrix with its *D*_*ii*_ equal to the sum of the *i*-th row of *W*. Let $F^{*}=\left[f_{1}^{*},\cdots \;,f_{l+u}^{*}\right]=\text{arg}\underset{\left\{f_{i}\right\}}{\min}L\left(\left\{f_{i}\right\}\right)$ be the optimal solution, it has been shown in Zhou et al. (2004) that

(3) $$\begin{array}{r} F^{*}={\left(\left(1+\lambda \right)I-\lambda D^{-1/2}WD^{-1/2}\right)}^{-1}Y. \end{array}$$

Based on the learned functions F^*^, we can predict the label information of *X*_*u*_ and then utilize such information to estimate more accurate information gain. Specifically, we let ${\hat{y}}_{u}$ denotes the predicted label of *X*_*u*_, and for node *S* we compute Gini index $G_{m}\left(S\right)=\overset{K}{\underset{k=1}{\sum }}p_{k}\left(1-p_{k}\right)$, where

$$p_{k}=\frac{1}{\left|S\right|}\left(\underset{x_{i}\in S,1\leq i\leq l}{\sum }{𝟙}_{\left\{{\left(y_{l}\right)}_{i}=k\right\}}+\underset{x_{i}\in S,l+1\leq i\leq l+u}{\sum }{𝟙}_{\left\{{\left({\hat{y}}_{u}\right)}_{i}=k\right\}}\right)$$

is the proportion of data from class *k*. Note that we utilize information from both labeled and unlabeled data to compute the Gini index. For each node, we estimate information gain as

(4) $$\begin{array}{r} G_{m}\left(w,\tau ,X_{l},Y_{l},X_{u}\right)=G_{m}\left(S\right)-\left(|S_{l}|G_{m}\left(S_{l}\right)+|S_{u}|G_{m}\left(S_{u}\right)\right)/|S|, \end{array}$$

where *S*_*l*_ and *S*_*u*_ are left and right child nodes, respectively.

## 4. Construction of Semi-Supervised Random Forest

In our framework, we preserve the major structure of the standard random forest where the testing stage is exactly the same as the standard one. As illustrated in the right part of Figure 1, we only make a small modification in stage 3 of Algorithm 1 where the splitting efficiency is now evaluated by our novel graph-embedded based information gain *G*_*m*_(τ_*j*_, *w*_*j*_, *X*_*l*_, *Y*_*l*_, *X*_*u*_) from Equation (4). Specifically, we leave stage 2 unchanged that the threshold τ of each subspace candidate *w* is still based on standard information gain such as the Gini index. Now with a set of parameter candidates *w*, τ, the stage 3 calculates the corresponding manifold based information score ĝ(*w*, τ) instead and select the optimal one through $\underset{w_{j},\tau_{j}}{\text{max}}ĝ\left(w_{j},\tau_{j}\right)$.

## 5. Experiments

We evaluate our method on both 2D, and 3D brain related medical image segmentation tasks as well as two machine learning benchmarks.

The retinal vessel, a part of the Central Nervous System (CNS), directly reflects the vascular condition of CNS. The accurate segmentation of vessels is important for this analysis. Much progress has been made based on either random forest (Gu et al., 2017) or deep learning (Liu et al., 2019). The DRIVE dataset (Staal et al., 2004) is a widely used 2D retinal vessel segmentation dataset that comprises of 20 training images and 20 testing ones. Each image is a 768 ×584 color image along with manual segmentation. For the image, we extract two types of widely used features: 1, local patch $x_{1}\in R^{15\times 15\times 3}$ of target. 2, $x_{2}\in R^{4\times 7\times 3}$ Gabor wavelets (Soares et al., 2006). We also investigate the single neuron segmentation in a brain image. BigNeuron project^1^ (Peng et al., 2015) is a 3D neuronal dataset with ground truth annotation from experts. For BigNeuron data, we manually picked 13 images among which a random 10 were used for training while the rest were left for testing, because this dataset is designed for tracing rather than segmentation. For example, some annotation is visibly thinner than the actual neuron. Furthermore, the image may contain multiple neurons but only one is properly annotated. For both datasets, we randomly collected 40, 000 (20, 000 positive and 20, 000 negative) samples from the training and testing sets, respectively. For 3D data, our feature is $x_{1}\in R^{15\times 15\times 7}$ local cube similar to the setting of Gu et al. (2017).

Apart from the medical imaging, we also demonstrate the generality of our method on two binary machine learning benchmark, IJCNN1 (Prokhorov, 2001) and Madelon (Guyon et al., 2004), in Libsvm Repository (Chang and Lin, 2011).

During the evaluation, we randomly selected a certain number *n* of labeled samples from the whole training set while leaving the rest unlabeled. Standard Random Forest (RF) is trained with *n* labeled training data only. Our method and RobustNode (Liu et al., 2015) are trained with both labeled data and unlabeled data. For reference, we also compared it with Optimal RF which is trained with labeled data as a standard RF. However, its node splitting is supervised with the whole training samples and their label. Optimal RF indicates the upper bound for all of semi-supervised learning algorithms.

### 5.1. Medical Imaging Segmentation

**Figure 3:**
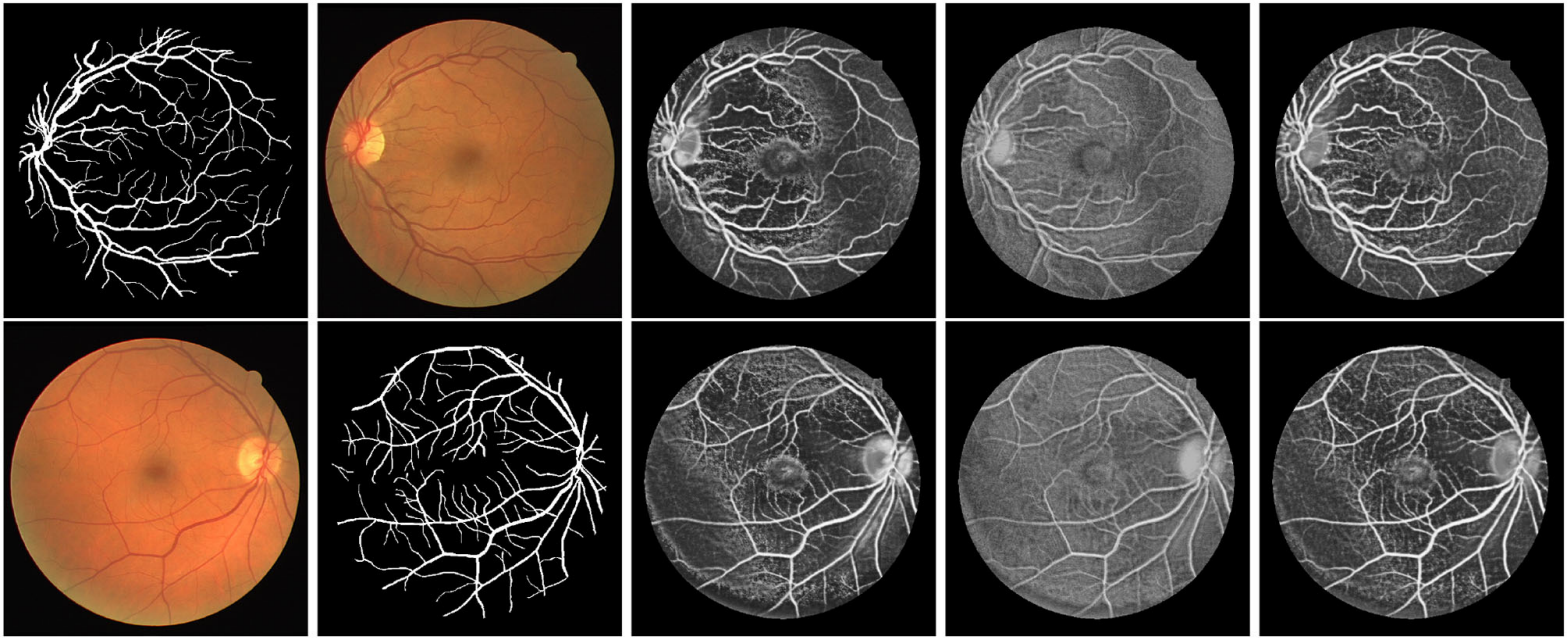
Exemplar estimation of vessel on the DRIVE dataset with 800 labeled samples. From left to right: Input images; Ground-truth; Estimation of our method; Estimation of Standard RF; Estimation of Optimal RF.

First, we illustrate the visual performance of segmentation in Figure 3. The estimated score is the possibility of the vessel given by the individual method. Our algorithm has consistently improved the estimation compared to the standard RF.

### 5.2. Quantitative Analysis

**Figure 4:**
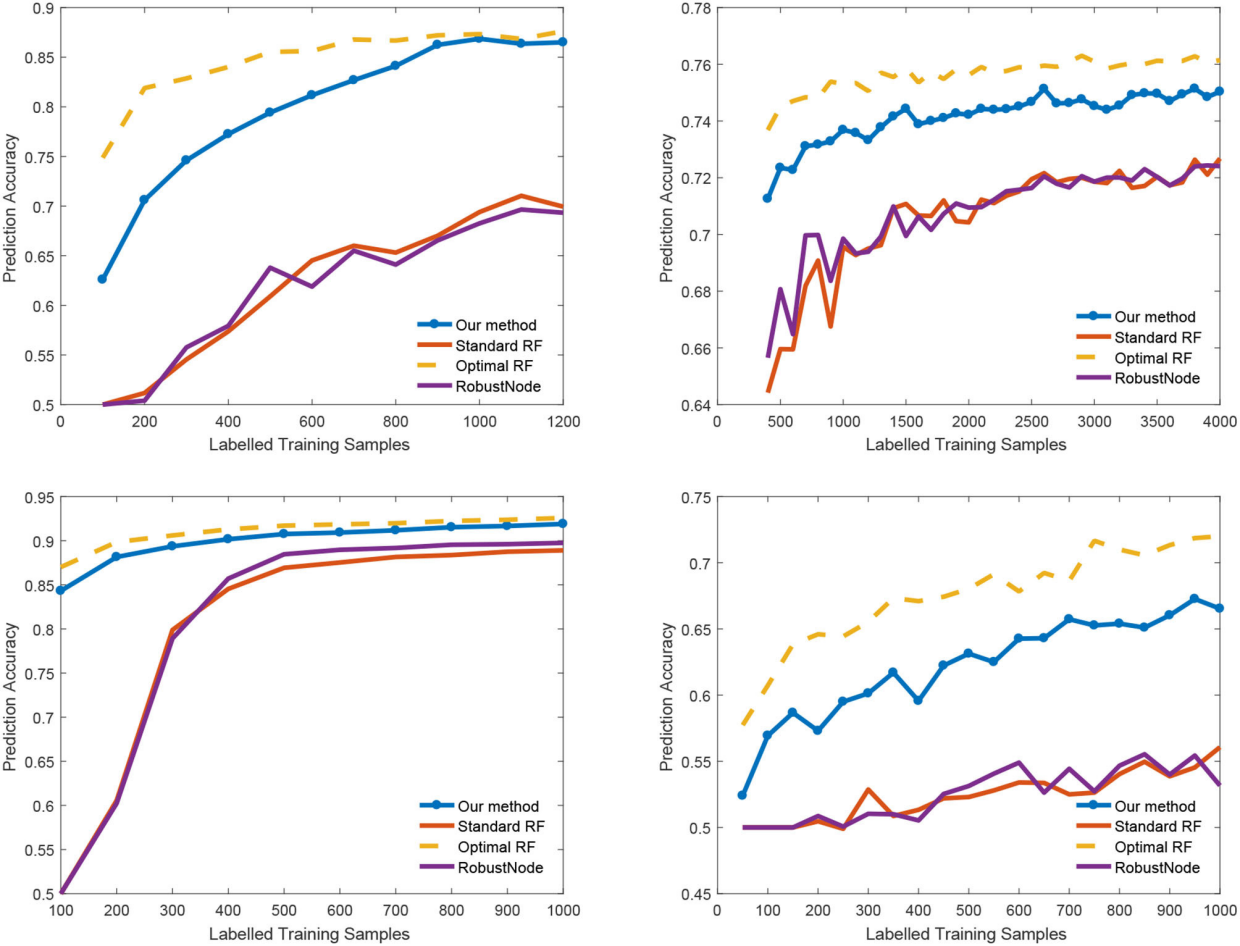
Classification accuracy vs. number of labeled samples.

We also report the classification accuracy with respect to the number of labeled data in Figure 4, Table 1. We compared our method with alternatives on both medical imaging segmentation and machine learning benchmarks. Figure 4 shows that our algorithm significantly outperformed alternative methods. Specifically, in the DRIVE dataset, our algorithm approaches the upper bound at 1,000 labeled samples. In the IJCNN1 dataset, our method quickly approaches the optimal one while the alternatives take 400 samples to approach.

**Table 1:** Classification accuracy (represented in percentage %) on different dataset.

	**Drive**	**Big neuron**	**IJCNN1**	**Madelon**
Our method	79.42	74.16	89.36	59.57
Standard RF	60.90	70.93	79.89	51.33
Robust node RF	63.79	70.99	78.92	50.53
Optimal RF	85.53	75.55	91.29	67.10

*We show the accuracy on the training sample of 400 (DRIVE), 1,500 (Big Neuron), 300 (IJCNN1), and 400 (Madelon)*.

## 6. Conclusion

In this paper, we propose a novel semi-supervised random forest to tackle the challenging problem of the lacking annotation in the analysis of medical imaging such as a brain image. Observing that the bottleneck of the standard random forest is the biased information gain estimation, we replaced it with a novel graph-embedded entropy which incorporates information from both labeled and unlabeled data. Empirical results show that our information gain is more reliable than the one used in traditional random forest under insufficient labeled data. By slightly modifying the training process of the standard random forest, our algorithm significantly improves the performance while preserving the virtue of the random forest. Our method has shown a superior performance with very limited data in both brain imaging analysis and machine learning benchmarks.

## Data Availability Statement

All datasets generated for this study are included in the article/supplementary material.

## Author Contributions

All authors listed have made a substantial, direct and intellectual contribution to the work, and approved it for publication.

## Conflict of Interest

The authors declare that the research was conducted in the absence of any commercial or financial relationships that could be construed as a potential conflict of interest.

## Footnotes

^1^https://www.alleninstitute.org/bigneuron/about/
